# Assessing Sustained B-Cell Depletion and Disease Activity in a French Multiple Sclerosis Cohort Treated by Long-Term IV Anti-CD20 Antibody Therapy

**DOI:** 10.1007/s13311-023-01446-5

**Published:** 2023-10-26

**Authors:** Sean A. Freeman, Bruno Lemarchant, Tifanie Alberto, Julie Boucher, Olivier Outteryck, Myriam Labalette, Stéphanie Rogeau, Sylvain Dubucquoi, Hélène Zéphir

**Affiliations:** 1 Department of Neurology, CRC-SEP, CHU of Lille, Lille, France; 2 Laboratory of Neuroinflammation and Multiple Sclerosis (NEMESIS), Univ. Lille, INSERM, CHU Lille, U1172 Lille, France; 3 Department of Neuroradiology, CHU Lille, Roger Salengro Hospital, Lille, France; 4 Univ. Lille, INSERM, CHU Lille, U1286 - INFINITE - Institute for Translational Research in Inflammation, Lille, France

**Keywords:** Multiple sclerosis, Hypogammaglobulinemia, Lymphopenia, NEDA, MEDA, Anti-CD20

## Abstract

Few studies have investigated sustained B-cell depletion after long-term intravenous (IV) anti-CD20 B-cell depleting therapy (BCDT) in multiple sclerosis (MS) with respect to strict and/or minimal disease activity. The main objective of this study was to investigate how sustained B-cell depletion after BCDT influences clinical and radiological stability as defined by “no evidence of disease activity” (NEDA-3) and “minimal evidence of disease activity” (MEDA) status in MS patients at 12 and 18 months. Furthermore, we assessed the frequency of serious adverse events (SAE), and the influence of prior lymphocytopenia-inducing treatment (LIT) on lymphocyte subset counts and gammaglobulins in MS patients receiving long-term BCDT. We performed a retrospective, prospectively collected, study in a cohort of 192 MS patients of all clinical phenotypes treated by BCDT between January 2014 and September 2021. Overall, 84.2% and 96.9% of patients attained NEDA-3 and MEDA status at 18 months, respectively. Sustained CD19^+^ depletion was observed in 85.8% of patients at 18 months. No significant difference was observed when comparing patients achieving either NEDA-3 or MEDA at 18 months and sustained B-cell depletion. Compared to baseline levels, IgM and IgG levels on BCDT significantly decreased at 6 months and 30 months, respectively. Patients receiving LIT prior to BCDT showed significant CD4^+^ lymphocytopenia and lower IgG levels compared to non-LIT patients. Grade 3 or above SAEs were rare. As nearly all patients achieved MEDA at 18 months, we suggest tailoring IV BCDT after 18 months given the occurrence of lymphocytopenia, hypogammaglobulinemia, and SAE after this time point.

**Supplementary Information:**

The online version contains supplementary material available at 10.1007/s13311-023-01446-5.

## Introduction

Multiple Sclerosis (MS) is a chronic, inflammatory demyelinating and neurodegenerative disease that affects the central nervous system and leads to variable degrees of physical and cognitive handicap. The success of phase II and III clinical trials using selective intravenous (IV) B-cell depleting therapies (BCDT) targeting B-lymphocyte CD20 antigen has changed the landscape of treating not only relapsing MS (RMS) [1–6], but also active progressive MS (PMS) [7].

BCDT in MS has shown persistent B-cell depletion over time from several months to over one year [1, 2, 8]. Yet, in contrast with other autoimmune diseases that are treated by IV BCDT, such as rituximab in rheumatoid arthritis using a “treat-to-target” approach [9], treatment of MS patients is based on a fixed-schedule dosing regimen every 6 months. Several groups have looked into spaced BCDT dosing intervals or lowering BCDT dosing, with few relapses and minimal MRI activity [10–15], despite the reemergence of a significant proportion of CD19^+^ B-cells [10]. Other studies have also tailored BCDT infusions in MS patients to either CD19^+^ B-cell or CD27^+^ memory B-cell repopulation [11, 16]. However, it is unknown whether reappearance of B-cells or B-cell subsets in MS is predictive of disease activity, similar to other neurological autoimmune disorders [17–19].

Long-term IV BCDT comes at a cost of increasing risk of severe infection [20–23]. Indeed, the duration of anti-CD20 therapy has been independently associated with a higher risk of infections requiring hospitalizations in MS [23, 24]. Furthermore, prior disease modifying therapies (DMT) before BCDT initiation in MS patients may influence T-lymphocyte populations as it has been seen recently that fingolimod can have a carry-over effect when administering ocrelizumab [25]. Taken together, the risks and benefits, as well as the patient profile, need to be weighed regarding long-term B-cell suppression.

To date, only few studies have studied B-cell repopulation kinetics after multiple cycles of IV BCDT in MS [15, 26, 27] and no study to date has compared sustained depletion to strict and/or minimal disease activity defined by objective measures used in prospective studies [28–30].

To improve the long-term strategy of IV BCDT therapy in MS, we aimed to investigate which parameters related to sustained B-cell depletion in MS patients (relapsing, primary and secondary progressive phenotypes) treated with IV anti-CD20 BCDT (rituximab and ocrelizumab) over at least one year could influence disease activity as defined by NEDA-3 and MEDA criteria and also the risk of developing serious adverse events.

## Methods

### Study Design and Population

We designed a monocentric, retrospective study based on data collected prospectively in the MS expert center of the University Hospital in Lille, France. The study was declared and data collection was performed in accordance with the recommendations of the French commission for data protection (CNIL) on November 30th, 2021.

In this study, we included patients with all clinical phenotypes of MS [RMS, active or non-active primary progressive MS (PPMS), and active or non-active secondary progressive MS(SPMS)] who started an anti-CD20 therapy (either ocrelizumab or off-label rituximab) based on clinical and/or radiological progression according to current health care authorization in France between January 2014 and September 2021 and with at least 3 cycles of BCDT. We excluded patients who did not undertake brain or spine MRI in the year prior to initiating BCDT. We collected demographic information including age and sex, as well as MS clinical history, which included date of first clinical symptoms, date of defined clinical MS with age of disease onset, disease duration, all clinical relapses (defined below), corticosteroid use for relapses, all prior disease modifying therapies (DMT), DMTs of which were highly-effective (natalizumab, mitoxantrone, fingolimod or alemtuzumab) or lymphocytopenia-inducing therapy (LIT) (mitoxantrone, fingolimod, siponimod, dimethyl fumarate, mycophenolate mofetil, methotrexate, cyclophosphamide, azathioprine and alemtuzumab), wash out time prior to BCDT, and date of introduction of BCDT. Data collected, when possible, while on BCDT included baseline and follow-up *Expanded Disability Status Scale* (EDSS) score every 6 months, annual brain and/or spinal MRI activity or radiological stability compared to the previous year’s MRI, and clinical relapses while on treatment (defined below).

Intravenous anti-CD20 treatment with ocrelizumab was administered based on fixed-scheduled dosing of 600 mg every 6 months after an initial dose of 600 mg divided into two injections spaced 2 weeks apart, while rituximab was administered based on two 1000 mg injections spaced 2 weeks apart followed by a maintenance dose of 1000 mg every 6 months.

### Primary Outcome and Secondary Objectives

The primary objective of this study was to compare NEDA-3 and MEDA status attainment at 12 and 18 months with respect to sustained B-cell depletion.

Sustained B-cell depletion was achieved when absolute CD19 counts were below 1% of the absolute CD45 count (total lymphocyte population). CD19 flow cytometry counts were performed, when possible, before each scheduled IV infusion of BCDT. Achieving NEDA-3 status was defined as the absence of clinical relapse (defined as new or recurrent neurological symptoms lasting more than 24 h without signs of fever or infection), absence of confirmed clinical disease progression (which was defined as an increase in EDSS by ≥ 1.5 if EDSS = 0, ≥ 1.0 if EDSS between 0 and 5.0, and ≥ 0.5 if EDSS ≥ 5.5 after two consecutive neurological examinations at least 6 months apart), and absence of activity on either annual brain or spinal MRI (with activity defined as an increase in T2 lesion size or number, or T1 gadolinium contrast-enhancement compared to the previous MRI) [28]. NEDA-3 activity was calculated at 12 months using the reference MRI [otherwise known as “re-baselined” MRI according to *L*’*Observatoire Français de la Sclérose en Plaques* (OFSEP) recommendations, i.e., at least 6 months after treatment introduction] compared to the MRI prior to BCDT initiation, while the MRI at 18 months was compared to the reference MRI.

MEDA achievement was defined by the absence of clinical activity and absence of focal MRI activity according to criteria by Prosperini and colleagues [30]. Briefly, clinical activity was defined as presence of clinical relapse with new or recurrent neurological symptoms lasting more than 24 h without signs of fever or infection. Focal MRI activity corresponded to contrast-enhancing lesions or new T2-lesions (≥ 3 new lesions). MEDA MRI activity at 12 and 18 months was compared similarly to NEDA-3.

Secondary objectives were to investigate lymphocyte subsets and natural killer (NK) cell counts at baseline and before every scheduled 6-month IV BCDT, to detail IgM, IgG and IgA levels, and to report serious adverse events under BCDT. Further secondary objectives looked to evaluate the number of grade-3 serious adverse events according to the Common Terminology Criteria for Adverse Events v4.03 as needing IV therapy or hospitalization while on BCDT. We collected data, when available, on length of hospital stay, clinical outcome and whether anti-CD20 therapy was continued or not. We also collected last Ig levels and CD19^+^ counts prior to infection. We additionally investigated whether previous LIT prior to BCDT could influence long-term T-cell population dynamics and Ig isotypes.

### Collection of Biological Data

We collected biological data, when possible, prior to the introduction of BCDT and every 6-months prior to each new infusion, which included total absolute lymphocyte counts by immunophenotyping using multi-color flow cytometry labeling for CD45^+^, and lymphocyte sub-populations defined by CD3^+^, CD4^+^ and CD8^+^ for T-cells, CD19^+^ for B-cells (surrogate marker for CD20^+^ depletion) and CD16^+^CD56^+^ for NK cells. Lymphocytopenia was defined as total lymphocyte and lymphocyte subsets at values inferior than the lower limit of normal (CD45^+^ < 1100 cells/mm^3^, CD3^+^ < 700 cells/mm^3^, CD4^+^ < 400 cells/mm^3^, CD8^+^ < 200 cells/mm^3^, CD19^+^ < 100 cells/mm^3^ and CD16^+^CD56^+^ < 100 cells/mm^3^). B cell depletion was defined as ≤ 1% of CD19^+^ cells of total CD45^+^ lymphocyte count. Total IgA, IgG and IgM isotype levels were also collected, with hypogammaglobulinemia defined as hypoIgA < 0.7 g/L, hypoIgM < 0.4 g/L and hypoIgG < 7.0 g/L.

### Statistical Analysis

Baseline demographic characteristics (baseline disease, MRI and biological data), follow-up clinical data after BCDT, absolute lymphocytes, lymphocyte subsets, IgA, IgM and IgG counts, lymphocytopenia and hypogammaglobulinemia of the study population were presented as mean ± SD for continuous data or numbers (percentages) for categorical data. Continuous data were analyzed for differences among groups using the Mann–Whitney *U* test for two groups or Kruskal–Wallis test for multiple groups, and categorical data were analyzed using the Fisher exact test.

NEDA-3 or MEDA status achievement at 12 and 18 months was expressed as numbers (percentages) and analyzed using the Fisher exact test. Characteristics of patients achieving NEDA-3 or MEDA at 18 months were reported as either mean ± SD or numbers (percentages) where appropriate, with corresponding Mann–Whitney *U* or Fisher exact test, respectively, for statistical analysis between groups. Furthermore, a Bonferroni correction was applied to control for multiple comparisons for NEDA-3 and MEDA activity at 12 and 18 months with respect to varying clinical or biological parameters.

Statistical significance was defined as two-tailed *p* < 0.05. Analysis of data and graph production were performed using GraphPad Prism^®^ software version 8 (San Diego, CA, USA). Additionally, this was an explorative study with no correction for multiple comparisons.

### Availability of Data and Materials

Anonymized patient data may be shared and made available by request from any investigator.

## Results

### Demographic, Disease, MRI and Biological Characteristics of the Patients at Baseline

A total of 192 MS patients received BCDT in our MS expert center for more than three cycles, of which 120 (62.5%) had RMS, 34 (17.7%) SPMS and 38 (19.8%) PPMS. Demographic data are summarized in Table 1. RMS patients presented a clinically more active disease at baseline with a higher annual relapse rate (ARR) than progressive MS (PMS) patients. EDSS scores were lower at baseline in RMS patients compared with PMS patients. The majority of MRI scans prior to treatment were active (81.2%). As expected, disease duration, age at BCDT initiation, wash out and median number of prior DMTs was higher in progressive patients.

**Table 1:** Demographic, disease, MRI and biological characteristics of the patients at baseline

**Baseline**	**Overall (N = 192)**	**Relapsing** **(N = 120)**	**Secondary Progressive** **(N = 34)**	**Primary Progressive** **(N = 38)**	***P*-value**
Age at disease onset—years	31.97 ± 10.53	28.82 ± 8.87	31.59 ± 10.72	42.26 ± 9.94	0.309
Sex ratio female—number (%)	121 (62.37)	78 (65.00)	23 (67.64)	21 (55.26)	0.4767
Disease duration—years	13.46 ± 8.75	11.06 ± 7.58	22.17 ± 9.31	13.25 ± 6.68	**< 0.0001**
Age at BCDT start—years	42.57 ± 11.69	37.19 ± 9.79	50.71 ± 8.04	52.29 ± 9.39	**< 0.0001**
Wash out from prior treatment—days	253.03 ± 508.33	128.39 ± 345.26	484.78 ± 630.87	461.71 ± 695.24	**< 0.0001**
Median number of prior DMTs (range)	1 (0–7)	1 (0–5)	2 (0–7)	1 (0–3)	**< 0.0001**
Treatment prior to BCDT (%)					
Treatment Naive	44 (22.92)	23 (19.17)	3 (8.82)	18 (47.37)	
Any beta interferon	12 (6.25)	7 (5.83)	3 (8.82)	2 (5.26)	
Glatiramer acetate	8 (4.17)	6 (5.00)	1 (2.94)	1 (2.63)	
Teriflunomide	29 (15.10)	21 (17.50)	6 (17.65)	2 (5.26)	
Dimethyl fumarate	20 (10.42)	17 (14.17)	2 (5.88)	1 (2.63)	
Biotin	2 (1.04)	0	2 (5.88)	0	
Azathioprine	1 (0.52)	0	1 (2.94)	0	
Mycophenolate Mofetil	11 (5.73)	1 (0.83)	3 (8.82)	7 (18.42)	
Methotrexate	2 (1.04)	1 (0.83)	1 (2.94)	0	
Siponimod	1 (0.52)	0	1 (2.94)	0	
Fingolimod	37 (19.27)	29 (24.17)	5 (14.71)	3 (7.89)	
Natalizumab	17 (8.85)	13 (6.77)	4 (11.76)	0	
Alemtuzumab	1 (0.52)	1 (0.83)	0	0	
Mitoxantrone	1 (0.52)	1 (0.83)	0	0	
Cyclophosphamide	6 (3.13)	0	2 (5.88)	4 (10.53)	
ARR previous year	0.47 (0.73)	0.63 (0.81)	0.31 (0.53)	0.06 (0.23)	**< 0.0001**
Median baseline EDSS (range)^a^	3.5 (0.0–8.0)	2.5 (0.0–6.5)	6.0 (2.0–6.5)	5.5 (2.0–8.0)	**< 0.0001**
Number of patients with MRI gadolinium enhancement (%)	117 (60.9)	78 (65.0)	22 (66.7)	17 (44.8)	0.073
Number of patients with new MRI T2-lesions (%)	39 (20.3)	20 (16.7)	5 (14.7)	14 (36.8)	**0.018**
Number of patients with stable MRI at baseline (%)	36 (18.8)	22 (18.3)	7 (20.6)	7 (18.4)	0.955
Total initial CD45^+^ lymphocyte count—mm3^b^	1721 ± 728.3	1711.9 ± 769.2	1635.9 ± 608.7	1841.5 ± 686.2	0.436
CD3^+b^	1310.3 ± 739.0	1263.6 ± 588.5	1188.0 ± 521.3	1396.2 ± 550.1	0.236
CD4^+c^	827.7 ± 397.9	796.4 ± 394.3	799.1 ± 341.4	976.8 ± 441.0	0.137
CD8^+b^	483.5 ± 242.6	495.0 ± 247.2	441.3 ± 236.0	475.4 ± 236.6	0.452
CD19^+d^	243.8 ± 188.7	252.3 ± 218.7	233.7 ± 109.8	224.0 ± 116.7	0.862
CD16^+^CD56^+e^	181.0 ± 92.0	176.2 ± 94.4	177.2 ± 79.0	192 ± 99.81	0.662
Number of patients with lymphocytopenia (%)					
CD45^+c^	29 (17.7)	23 (21.9)	2 (7.14)	4 (12.9)	0.141
CD3^+b^	21 (12.8)	17 (16.2)	2 (7.14)	2 (6.4)	0.223
CD4^+c^	17 (10.3)	16 (15.1)	0 (0.0)	1 (3.2)	**0.023**
CD8^+b^	17 (10.3)	10 (9.4)	2 (7.4)	5 (16.1)	0.481
CD19^+d^	19 (11.7)	13 (12.4)	4 (14.8)	2 (6.7)	0.596
CD16^+^CD56^+^ — mm3^e^	28 (17.4)	17 (16.3)	6 (22.2)	5 (16.6)	0.767
Ig levels—g/L					
IgA^f^	2.07 ± 0.75	2.04 ± 0.69	1.90 ± 0.83	2.35 ± 0.83	0.078
IgG^g^	10.33 ± 2.69	10.53 ± 2.67	9.36 ± 2.73	10.62 ± 2.63	0.071
IgM^f^	1.17 ± 0.77	1.21 ± 0.60	1.16 ± 1.33	1.04 ± 0.47	0.603
Number of patients with hypogammaglobulinemia (%)					
IgA^f^	3 (1.81)	2 (1.9)	1 (3.3)	0 (0.0)	0.62
IgG^g^	8 (4.7)	3 (2.8)	3 (10.0)	2 (6.7)	0.229
IgM^f^	7 (4.2)	6 (5.6)	1 (3.3)	0 (0.0)	0.382

*p*-values < 0.05 were indicated in bold

^a^A total number of 176 patients had recorded initial EDSS scores, of which 112 relapsing, 30 secondary progressive and 34 primary progressive

^b^Total number of patients with CD45^+^, CD3^+^ and CD8^+^ flow cytometry counts for overall, relapsing, secondary progressive and primary progressive is 165, 106, 31 and 28, respectively

^c^Total number of CD4^+^ flow cytometry counts for overall, relapsing, secondary progressive and primary progressive is 152, 97, 29 and 26, respectively

^d^Total number of CD19^+^ flow cytometry counts for overall, relapsing, secondary progressive and primary progressive is 162, 95, 28 and 27, respectively

^e^Total number of CD16^+^ CD56^+^ flow cytometry counts for overall, relapsing, secondary progressive and primary progressive is 148, 105, 30 and 25, respectively

^f^Total number of IgA and IgM counts for overall, relapsing, secondary progressive and primary progressive is 156, 99, 29 and 28, respectively

^g^Total number of IgG and total Ig counts for overall, relapsing, secondary progressive and primary progressive is 157, 100, 29 and 28, respectively

We observed no significant differences between MS phenotype and mean total lymphocyte or lymphocyte subsets at baseline. CD45^+^ lymphocytopenia was observed in 17.7% of patients. Significant differences between lymphocytopenia in lymphocyte subsets and MS phenotype was only observed with regards to CD4^+^ T cells in RMS patients. There were no differences in mean Ig isotype levels between the MS phenotypes. Hypogammaglobulinemia was present for all isotypes at baseline, although this represented a small percentage of patients in the cohort (1.8% IgA, 4.2% IgM and 4.7% IgG).

### Clinical Follow up of the Patients after Receiving BCDT

The mean overall BCDT duration was 2.75 ± 1.30 years, with a mean number of 5.23 ± 2.14 perfusions. The mean overall perfusion interval was 196.2 ± 91.5 days and was different (*p* < 0.0001) before and after March 2020 (beginning of COVID-19 pandemic in France).

Further clinical follow up data is summarized in Table 2. The ARR at 12 and 18 months for the entire cohort was 0.05 ± 0.24 and 0.02 ± 0.16, respectively, which was lower than the ARR in the year prior to BCDT initiation for both time points (*p* < 0.0001). Overall, 60.6% and 84.2% of patients achieved NEDA-3 status at 12 and 18 months, respectively. At 12 months, MEDA status was reached in 84.6% of patients in our cohort, and 96.9% at 18 months. Of note, failure of NEDA-3 at 12 months was primarily due to MRI activity, with 75.3% of patients not achieving NEDA-3 at 12 months showing either new/enlarged T2 lesions or gadolinium enhancement. Of the patients with MRI activity, 4% showed gadolinium enhancement. NEDA-3 failure at 18 months was due to MRI activity in only 18% of at this timepoint. With regards to MEDA, failure to achieve MEDA at 12 months was due to MRI activity in 75.0% of patients, while failure of MEDA at 18 months was accounted for entirely with either confirmed clinical disease progression (2 patients) or clinically defined relapse (2 patients).

**Table 2:** Follow up of the patients after receiving anti-CD20 therapy

**Follow-up**	**Overall (N = 192)**	**Relapsing** **(N = 120)**	**Secondary Progressive** **(N = 34)**	**Primary Progressive** **(N = 38)**	***P*-value**
Treatment duration—years	2.75 ± 1.30	2.71 ± 1.44	2.95 ± 1.28	3.10 ± 1.00	**0.022**
Mean number of perfusions	5.23 ± 2.14	5.00 ± 2.25	5.39 ± 1.93	5.78 ± 1.84	**0.019**
ARR at 12 months	0.05 ± 0.24	0.06 ± 0.28	0.03 ± 0.17	0.02 ± 1.6	0.62
ARR at 18 months	0.02 ± 0.16	0.04 ± 0.20	0.00 ± 0.00	0.00 ± 0.00	0.239
Number of Pregnancies (%)	10	10 (100)	0 (0.0)	0 (0.0)	
Perfusion Interval—days					
Overall	196.2 ± 91.5	190 ± 28.9	194.9 ± 40.7	196.1 ± 42.2	0.074
Before March 2020	196.7 ± 44.7	191.5 ± 38.8	204.1 ± 60.7	199.3 ± 49.4	0.119
After March 2020	189.7 ± 24.7	189 ± 20.9	188.7 ± 14.5	192.7 ± 32.9	0.662
*P* value Before vs After March 2020	**< 0.0001**	0.246	**0.013**	**0.002**	
Attained NEDA-3 status at 12 months^a^—number (%)	100 (60.6)	60 (56.6)	19 (67.8)	21 (67.7)	0.369
Attained MEDA status at 12 months^b^—number (%)	154 (84.6)	94 (81.0)	28 (87.5)	32 (94.1)	0.156
Attained NEDA-3 status at 18 months^c^—number (%)	96 (84.2)	58 (82.8)	14 (82.3)	24 (88.8)	0.746
Attained MEDA status at 18 months^d^—number (%)	129 (96.9)	76 (95.0)	22 (100.0)	31 (100.0)	0.1505

*p*-values < 0.05 were indicated in bold

^a^A total of 165 patients had available data to analyze NEDA-3 status at 12 months, of which 106 were relapsing, 28 were secondary progressive and 31 were primary progressive

^b^A total of 182 patients had available data to analyze MEDA status at 12 months, of which 116 were relapsing, 32 were secondary progressive and 34 were primary progressive

^c^A total of 114 patients had available data to analyze NEDA-3 status at 18 months, of which 70 were relapsing, 17 were secondary progressive and 27 were primary progressive

^d^A total of 133 patients had available data to analyze MEDA status at 18 months, of which 80 were relapsing, 22 were secondary progressive and 31 were primary progressive

Taken together, these results suggest that a larger proportion of patients achieve strict disease control with ensuing cycles of BCDT, and that a higher proportion of patients at similar time points achieve disease control on BCDT when tolerating less stringent clinical and radiological thresholds.

### Sustained B-Cell Depletion Does Not Influence NEDA-3 and MEDA Status at 12 or 18 Months

We looked to explore the relationship between baseline demographic and clinical characteristics with respect to MS disease control using NEDA-3 or MEDA criteria. The results are summarized in Table 3. We observed no differences in univariate analysis between attaining NEDA-3 or MEDA status at 12 and 18 months when comparing age, disease duration, EDSS at baseline, MS phenotype, previous highly-effective treatment or naive to treatment.

**Table 3:** Comparison of demographic, clinical and biological characteristics between patients who did or did not achieve NEDA-3 or MEDA status at 12 and 18 months

	**NEDA-3 12 Months**			**NEDA-3 18 Months**			**MEDA 12 Months**			**MEDA 18 Months**		
	**NEDA-3** **(*n* = 100)**	**Non NEDA-3** **(*n* = 65)**	***P*-value**	**NEDA-3** **(*n* = 96)**	**Non NEDA- 3 (*n* = 18)**	***P*-value**	**MEDA** **(*n* = 154)**	**Non MEDA** **(*n* = 28)**	***P*-value**	**MEDA** **(*n* = 129)**	**Non MEDA (*n* = 4)**	***P*-value**
**Age—years**	44.10 ± 12.22	39.45 ± 11.00	0.475	44.36 ± 11.75	39.06 ± 9.72	> 0.9	43.81 ± 11.75	36.50 ± 9.08	0.05	43.7 ± 11.3	35.75 ± 9.53	> 0.9
**Disease Duration—years**	12.92 ± 8.58	12.85 ± 8.06	> 0.9	13.02 ± 8.24	11.52 ± 5.75	> 0.9	13.82 ± 8.75	11.23 ± 7.66	> 0.9	13.7 ± 8.36	8.44 ± 6.64	> 0.9
**EDSS at Baseline**	3.52 ± 1.97	3.50 ± 2.00	> 0.9	3.80 ± 1.91	4.13 ± 1.97	> 0.9	3.62 ± 1.96	2.98 ± 2.00	> 0.9	3.78 ± 1.87	4.167 ± 1.60	> 0.9
**Female Sex—number (%)**	61 (61.0)	39 (60.0)	> 0.9	54 (56.2)	13 (72.2)	> 0.9	95 (61.7)	19 (67.8)	> 0.9	77 (59.7)	3 (75)	> 0.9
**Phenotype—number (%)**			> 0.9			> 0.9			> 0.9			> 0.9
**Relapsing**	60 (60.0)	46 (70.7)	> 0.9	58 (60.4)	12 (66.7)	> 0.9	94 (61.0)	22 (78.6)	> 0.9	76 (58.9)	4 (100)	> 0.9
**Progressive**	40 (40.0)	19 (29.2)	> 0.9	38 (39.6)	6 (33.3)	> 0.9	60 (39.0)	6 (21.4)	> 0.9	53 (41.0)	0 (0.0)	> 0.9
**Previous highly efficacious treatment—number (%)**	38 (38.0)	29 (44.6)	> 0.9	21 (21.88)	3 (16.7)	> 0.9	38 (38.0)	29 (44.6)	> 0.9	52 (40.3)	1 (25.0)	> 0.9
**Naive to all treatment—number (%)**	25 (25)	13 (20.0)	> 0.9	36 (37.5)	8 (44.4)	> 0.9	34 (22.1)	6 (21.43)	> 0.9	29 (22.5)	1 (25.0)	> 0.9
**Mean flow cytometry counts— mm3**	**NEDA-3** **(n = 92)**^**a**^	**Non NEDA-3** **(n = 55)**	***P*-value**	**NEDA-3** **(n = 88)**	**Non NEDA-3** **(n = 15)**	***P*-value**	**MEDA** **(n = 134)**^**b**^	**Non MEDA** **(n = 22)**	***P*-value**	**MEDA** **(n = 113)**^**c**^	**Non MEDA** **(n = 2)**	***P*-value**
**CD45**^**+**^	1504 ± 993.0	1390 ± 592.9	> 0.9	1441 ± 483.5	1390 ± 592.9	> 0.9	1465 ± 900.1	1520 ± 445.8	> 0.9	1435 ± 500.3	1222 ± 82.0	> 0.9
**CD3**^**+**^	1180 ± 490.4	1184 ± 511.9	> 0.9	1182 ± 471.7	1049 ± 395.93	> 0.9	1171 ± 504.5	1320 ± 419.6	> 0.9	1181 ± 488.2	1069 ± 122.3	> 0.9
**CD4**^**+**^	806.6 ± 350.8	802.4 ± 385.3	> 0.9	824.2 ± 319.4	802.4 ± 385.3	> 0.9	792.3 ± 360.7	922.0 ± 326.2	> 0.9	809 ± 329.7	648 ± 118.1	> 0.9
**CD8**^**+**^	461.8 ± 234.6	464.3 ± 257.4	> 0.9	472.2 ± 244.9	441.1 ± 189.8	> 0.9	470.5 ± 254.2	456.3 ± 167.6	> 0.9	476 ± 238.6	445 ± 69.3	> 0.9
**CD16**^**+**^**/CD56**^**+**^	205.0 ± 130.9	191.1 ± 101.4	> 0.9	203.3 ± 128.2	197.7 ± 85.0	> 0.9	209.7 ± 124.4	164.7 ± 79.9	> 0.9	200 ± 121.8	134 ± 33.9	> 0.9
**CD19**^**+**^	9.2 ± 22.8	15.04 ± 27.5	> 0.9	8.38 ± 17.9	27.47 ± 89.44	> 0.9	9.9 ± 22.2	17.6 ± 33.1	> 0.9	11.4 ± 36.9	2.5 ± 2.1	> 0.9
**Lymphopenia—number (%)**	**NEDA-3** **(n = 92)**^**a**^	**Non NEDA-3** **(n = 59)**	***P*-value**	**NEDA-3** **(n = 88)**	**Non NEDA-3** **(n = 15)**	***P*-value**	**MEDA** **(n = 134)**^**b**^	**Non MEDA** **(n = 22)**	***P*-value**	**MEDA** **(n = 113)**^**c**^	**Non MEDA** **(n = 2)**	***P*-value**
**CD45**^**+**^	30 (31.5)	23 (38.9)	> 0.9	21 (23.8)	5 (31.2)	> 0.9	44 (32.8`)	4 (18.2)	> 0.9	29 (25.6)	0 (0.0)	> 0.9
**CD3**^**+**^	15 (15.7)	11 (18.6)	> 0.9	15 (17.0)	3 (18.7)	> 0.9	21 (15.7)	0 (0.0)	> 0.9	19 (16.8)	0 (0.0)	> 0.9
**CD4**^**+**^	10 (10.5)	10 (16.9)	> 0.9	4 (4.5)	2 (12.5)	> 0.9	14 (10.4)	1 (4.55)	> 0.9	7 (6.2)	0 (0.0)	> 0.9
**CD8**^**+**^	9 (9.4)	8 (13.5)	> 0.9	8 (9.0)	1 (6.2)	> 0.9	9 (6.6)	2 (9.1)	> 0.9	8 (7.1)	0 (0.0)	> 0.9
**CD16**^**+**^**/CD56**^**+**^	21 (22.3)	9 (15.2)	> 0.9	12 (13.6)	3 (18.7)	> 0.9	21 (15.7)	3 (14.2)	> 0.9	16 (14.3)	0 (0.0)	> 0.9
	**NEDA-3** **(n = 90)**	**Non NEDA-3** **(n = 54)**	***P*-value**	**NEDA-3** **(n = 88)**	**Non NEDA-3** **(n = 15)**	***P*-value**	**MEDA** **(n = 130)**	**Non MEDA** **(n = 22)**	***P*-value**	**MEDA** **(n = 107)**	**Non MEDA** **(n = 2)**	***P*-value**
**CD19**^**+**^** depleted—number (%)**	76 (84.4)	40 (74.0)	> 0.9	74 (85.0)	13 (86.6)	> 0.9	107 (82.3)	17 (77.2)	> 0.9	87 (81.3)	2 (100.0)	> 0.9
**Mean immunoglobulin levels—g/L**	**NEDA-3** **(n = 96)**	**Non NEDA-3** **(n = 58)**	***P*-value**	**NEDA-3** **(n = 92)**	**Non NEDA-3** **(n = 15)**	***P*-value**	**MEDA** **(n = 138)**	**Non MEDA** **(n = 24)**	***P*-value**	**MEDA** **(n = 117)**	**Non MEDA** **(n = 3)**	***P*-value**
**IgA**	1.98 ± 0.81	2.21 ± 0.85	> 0.9	1.94 ± 0.79	2.02 ± 0.89	> 0.9	2.03 ± 0.82	2.23 ± 0.81	> 0.9	1.96 ± 0.83	2.37 ± 0.61	> 0.9
**IgM**	0.83 ± 1.33	0.72 ± 0.48	> 0.9	0.72 ± 0.97	0.59 ± 0.35	> 0.9	0.80 ± 1.13	0.61 ± 0.40	> 0.9	0.69 ± 0.87	0.90 ± 0.33	> 0.9
**IgG**	9.62 ± 2.61	10.26 ± 2.46	> 0.9	9.66 ± 2.68	8.97 ± 1.91	> 0.9	9.82 ± 2.57	10.2 ± 2.53	> 0.9	9.52 ± 2.45	12.2 ± 1.98	> 0.9
**Hypogammaglobulinemia—number (%)**	**NEDA-3** **(n = 96)**	**Non NEDA-3** **(n = 58)**	***P*-value**	**NEDA-3** **(n = 92)**	**Non NEDA-3** **(n = 15)**	***P*-value**	**MEDA** **(n = 138)**	**Non MEDA** **(n = 24)**	***P*-value**	**MEDA** **(n = 117)**	**Non MEDA** **(n = 3)**	***P*-value**
**IgA**	4 (4.16)	5 (8.62)	> 0.9	6 (6.52)	0 (0.00)	> 0.9	5 (3.6)	0 (0.0)	> 0.9	4 (3.4)	0 (0.0)	> 0.9
**IgM**	18 (18.75)	15 (25.86)	> 0.9	28 (30.43)	5 (33.33)	> 0.9	25 (18.1)	7 (29.1)	> 0.9	33 (28.4)	0 (0.0)	> 0.9
**IgG**	10 (10.42)	4 (6.90)	> 0.9	9 (9.78)	1 (6.25)	> 0.9	14 (10.1)	1 (4.1)	> 0.9	11 (9.2)	0 (0.0)	> 0.9

^a^Total number of available data for CD45^ +^, CD3^+^, CD4^+^ and CD8^+^ cell counts are 92, and available data for CD16^+^ /CD56^+^ and CD19^+^ cell counts are 91

^b^Total number of available data for CD45^+^ and CD3^+^ are 133, CD4^+^ and CD8^+^ cell counts are 134, CD16^+^ /CD56^+^ cell counts are 132, and CD19^+^ cell counts are 131

^c^Total number of available data for CD45^+^, CD3^+^, CD4^+^ and CD8^+^ cell counts are 113, and available data for CD16^+^ /CD56^+^ and CD19^+^ cell counts are 112

We further explored the relationship between clinical and radiological control of disease activity defined by either NEDA-3 or MEDA criteria and sustained depletion of circulating CD19^+^ lymphocytes after BCDT. These results are also summarized in Table 3. We observed no differences in comparing sustained B-cell depletion or repopulation and NEDA-3 or MEDA status at 12 months or 18 months. We found no difference in mean absolute values of total CD45^+^ lymphocytes, CD3^+^, CD4^+^, CD8^+^ or CD16^+^56^+^ and NEDA-3 or MEDA status at 12 and 18 months. No differences were observed in comparing lymphocytopenia and NEDA-3 or MEDA status at 12 and 18 months. Additionally, no differences were observed when comparing mean immunoglobulin levels or hypogammaglobulinemia status in Ig isotypes and NEDA-3 or MEDA status at 12 or 18 months.

These results suggest that sustained B-cell depletion status does not predict achievement of clinical and radiological stability at 12 and 18 months using accepted scores for disease control.

### Lymphocytopenia and Hypogammaglobulinemia after BCDT

**Fig. 1:**
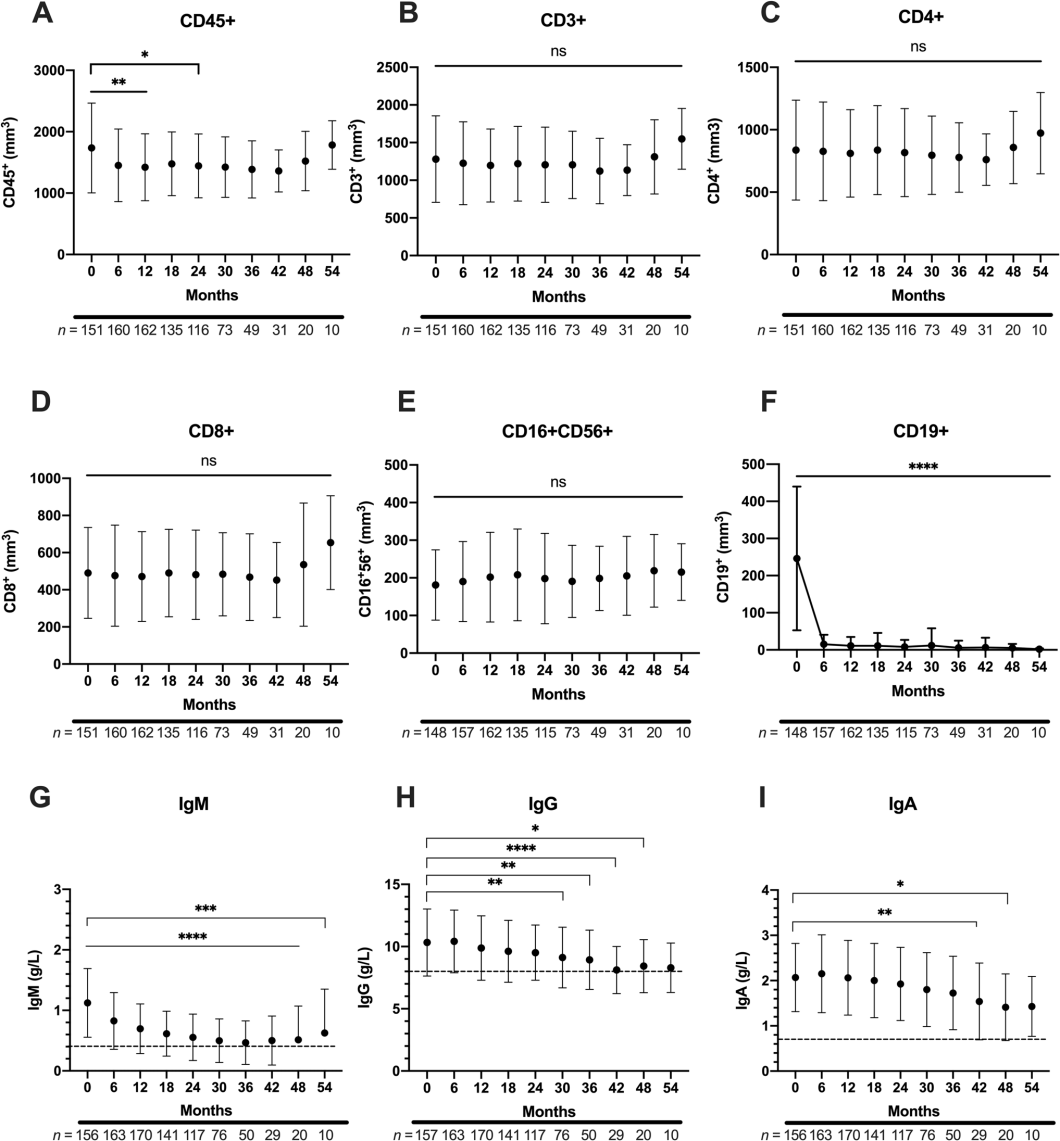
Mean total lymphocyte subset counts and immunoglobulin levels in the patient cohort. Mean total lymphocyte (**A**) and lymphocyte subsets (**B**–**F**), as well as mean immunoglobulin levels (**G**–**I**) were analyzed from baseline (0 months) until 54 months. Compared to baseline, a significant reduction in the absolute CD45^+^ lymphocyte count was observed at 6, 12, and 24 months (**A**); however, no significant reduction was observed for T-lymphocyte subsets or NK cells (**B**–**E**). An expected significant decrease in the mean CD19^+^ population was observed post-BCDT (**F**). Lower IgA levels were observed at 42 and 48 months compared to baseline (**G**), while lower IgG levels were observed between 30 and 48 months post-BCDT (**H**). Lower IgM levels were observed starting at 6 months post-BCDT and persisted until 54 months post-BCDT (**I**). Dotted line denotes hypogammaglobulinemia threshold. Number of patients analyzed is detailed below each month. Error bars represent standard error of the mean. Statistical analysis by Kruskal–Wallis tests with significant *p* values denoted by * for *p* < 0.05, ** for* p* < 0.01, and *** for *p* < 0.001

Follow up of patients receiving BCDT showed a decrease in the mean total lymphocyte count at 6, 12 and 24 months when compared to baseline total lymphocyte counts (Fig. 1A; *p* = 0.004, *p* = 0.001 and *p* = 0.04, respectively). In line with this observation, we did observe an increase in the percentage of patients presenting with sustained CD45^+^ lymphocytopenia at six months (31.9%), which remained stable after subsequent injections up to 42 months. There was no significant difference in mean CD3^+^, CD4^+^, CD8^+^ or CD16^+^CD56^+^ cells when compared to baseline (Fig. 1B–E). The percentage of patients with sustained lymphocytopenia in these lymphocyte subsets remained stable throughout the observation period (Supplemental Fig. 1A). As expected, we observed a sustained depletion of CD19^+^ cells from 6 to 54 months when compared to baseline (*p* < 0.0001) (Supplemental Fig. 1A). With the cumulative effect of BCDT over time, the percentage of patients with persistent B-cell depletion progressively increased over the ensuing perfusion cycles (73.2% depleted at six months to 96.7% at 42 months).

In our cohort, IgM levels were lower at all time points compared to baseline starting at 6 months of treatment (*p* = 0.0001) (Fig. 1G). Furthermore, we observed lower IgG levels starting at 30 months of BCDT when compared to baseline (*p* = 0.003), (Fig. 1H). IgA levels were reduced at 42 months (*p* = 0.008) when compared to baseline (Fig. 1I). The percentage of patients with hypogammaglobulinemia increased gradually in all isotypes with an increasing number of perfusions (Supplemental Fig. 1B).

These results support previous studies that long-term BCDT can lead to hypogammaglobulinemia of all isotypes with about one third of patients having hypoIgG at 42 months of BCDT, and that subsequent cycles of BCDT does not lead to significant differences in T-cell lymphocyte subsets when compared to baseline.

### Previous Lymphocytopenia Inducing Treatments (LIT) Prior to BCDT Influences T-Cell Subsets and Ig Isotype Dynamics

**Fig. 2:**
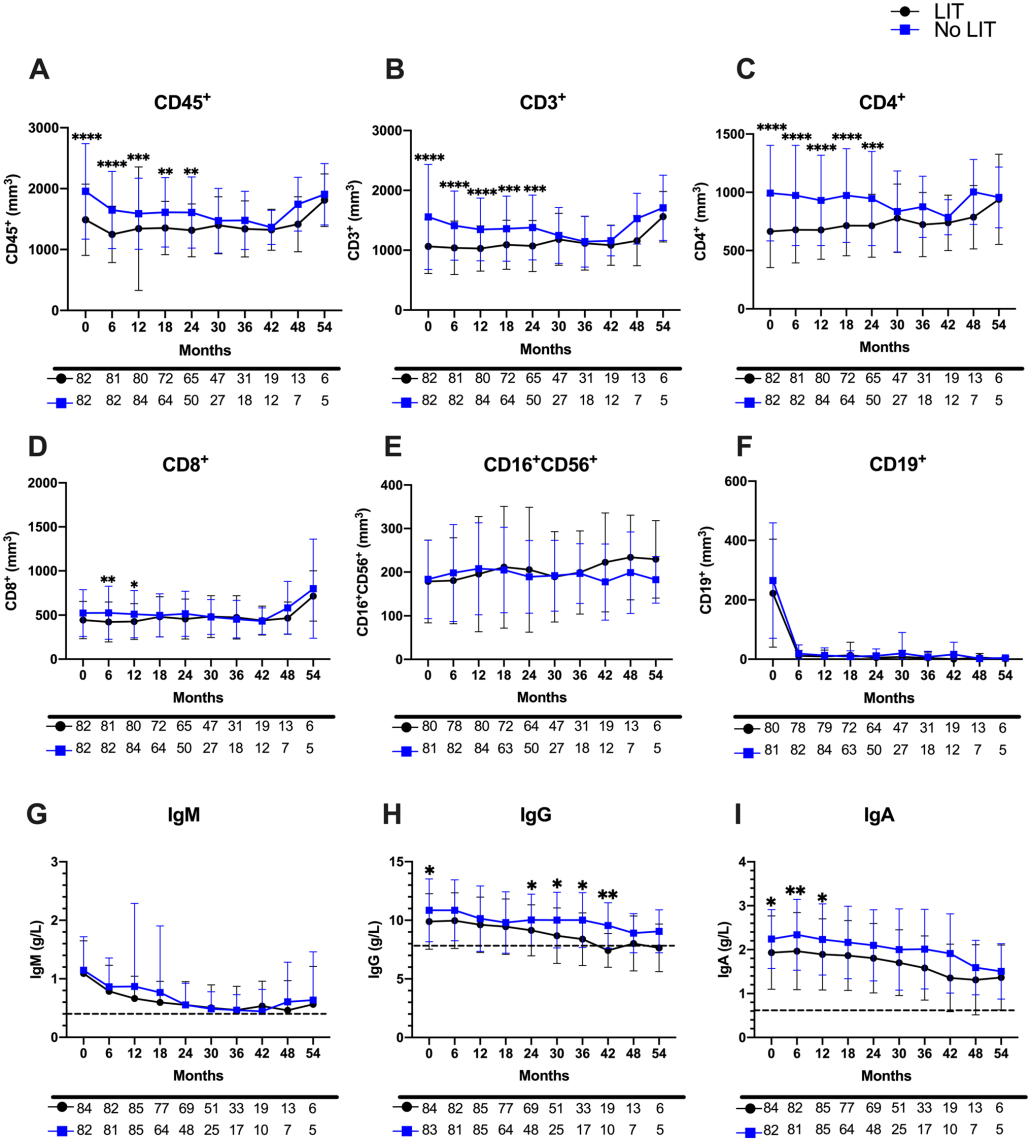
Prior lymphocytopenia-inducing treatment (LIT) and mean subset lymphocyte counts and IgG levels of the cohort treated by BCDT. Mean total lymphocyte (**A**) and lymphocyte subsets (**B**–**F**), as well as mean immunoglobulin levels (**G**–**I**) were analyzed from baseline (0 months) until 54 months in patients having received (black line and black circles) or not received prior LIT (blue line and blue squares). Initial mean CD45^+^, CD3^+^ and CD4^+^ lymphocyte counts are significantly decreased in patients having received prior LIT compared to those not having received prior LIT, which also persists until 24 months post-BCDT (**A**–**C**). Mean CD8^+^ counts are significantly different only at 6 and 12 months post-BCDT when comparing these two populations (**D**). No differences were observed for NK cells or CD19^+^ depletion (**E** and **F**). No differences were observed in IgM levels when comparing prior LIT and no prior LIT (**G**). Mean IgG levels were significantly different at baseline and from 24 to 42 months when comparing these two groups (**G**), and additionally, mean IgA levels were significantly different between the two groups for the first 12 months (**I**). Dotted line denotes hypogammaglobulinemia. Number of patients analyzed is detailed below each month. Error bars represent standard error of the mean. Statistical analysis by Mann Whitney *U* tests with significant *p* values denoted by * for *p* < 0.05, ** for* p* < 0.01, and *** for *p* < 0.001

**Fig. 3:**
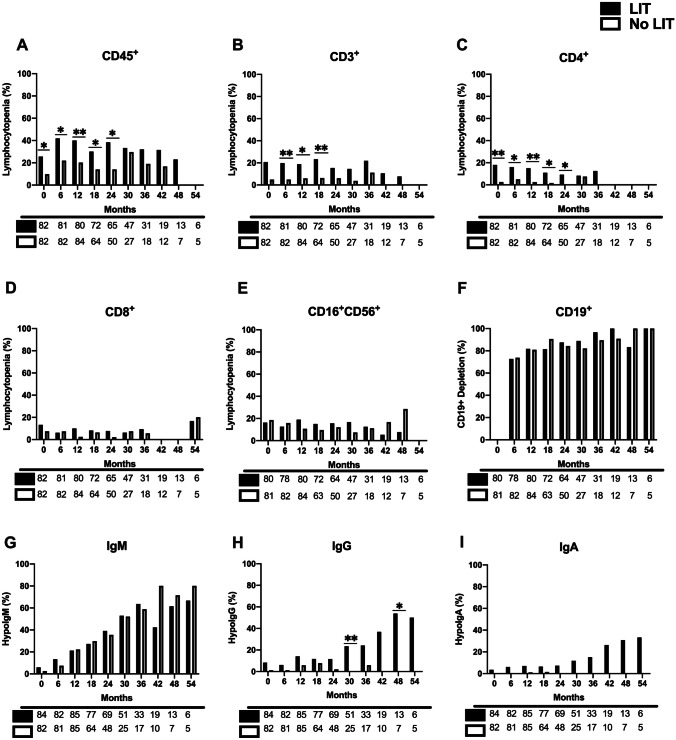
Prior lymphocytopenia-inducing treatment (LIT) and percentage lymphocytopenia and hypogammaglobulinemia in the patient cohort treated by BCDT. Comparison in the percentage of patients with lymphocytopenia and percentage of patients presenting with CD19^+^ depletion (**A**–**F**), as well as hypogammaglobulinemia (**G**–**I**), from baseline to 54 months in patients having received or not received prior LIT. A significant increase in the percentage of CD45^+^ and CD4^+^ lymphocytopenia was observed from baseline to 24 months in LIT patients post-BCDT (**A**, **C**), while there was an increase in the percentage of CD3^+^ lymphocytopenia in LIT patients from 6 to 18 months (**B**). No significant differences were observed for CD8^+^ lymphocytes, CD16^+^/CD56^+^ NK cells or CD19^+^ depletion (**D**–**F**). No differences in the percentage of IgA or IgM hypogammaglobulinemia was observed between the two groups, but hypoIgG was significantly increased at 30 and 48 months between prior LIT and no prior LIT patients. Number of patients analyzed is detailed below each month. Statistical analysis by Fisher exact tests with significant *p* values denoted by * for *p* < 0.05, ** for* p* < 0.01, and *** for *p* < 0.001

At baseline, we observed lower mean absolute CD45^+^, CD3^+^ and CD4^+^ lymphocyte counts when comparing patients with and without prior LIT (*p* < 0.0001 for total lymphocytes, CD3^+^ and CD4^+^ subsets) (Fig. 2A–C). The differences remained statistically significant between the two groups until 24 months for these three lymphocyte subsets, with significant difference for CD8^+^ lymphocytes appearing at 6 and 12 months (Fig. 2D). No differences were shown in mean absolute CD16^+^CD56^+^ or CD19^+^ counts between the two groups (Fig. 2E–F). Furthermore, a higher percentage of patients with CD45^+^ and CD4^+^ lymphocytopenia in the LIT group from baseline (*p* = 0.04 and *p* = 0.009, respectively) to 24 months of BDCT was observed (*p* = 0.036 and *p* = 0.033, respectively) (Fig. 3A, C). The percentage of patients with CD3^+^ lymphocytopenia was different from 6 to 18 months (*p* = 0.007) between the two groups (Fig. 3B).

There was a difference in mean IgA levels at baseline, 6 and 12 months between these two groups (*p* = 0.010, *p* = 0.008 and *p* = 0.012, respectively) (Fig. 2G, I). Mean IgG levels were different at baseline, and from 24 to 42 months between the two groups (Fig. 2H). No differences were observed with respect to mean IgM levels throughout the study period. We only observed a difference in the percentage of patients with IgG hypogammaglobulinemia at 30 and 48 months (*p* = 0.007 and *p* = 0.044, respectively) (Fig. 3I).

Taken together, these results suggest that prior LIT preceding BCDT introduction seems to play a role in initial T-lymphocytopenia from baseline and until 24 months, while previous LIT appears to significantly induce IgG hypogammaglobulinemia after 4 cycles of BCDT.

### Risk of Serious Adverse Events are Rare on BCDT

We observed 21 grade-3 or above serious adverse events (SAE), with 4 patients presenting more than 2 SAEs. The overall SAE rate was 4.04/100 patient-years. SAE were mainly infections, with the largest proportion being urinary tract infection (42.8%). Results are further presented in Table 4. The mean number of injections prior to SAE were 4.45 ± 2.24. Only one patient discontinued BCDT permanently and no patients deceased on BCDT during the study period.

**Table 4:** Serious adverse events (SAE) and population characteristics prior to SAE

	**Overall**	**Relapsing**	**Secondary Progressive**	**Primary Progressive**
Grade-3 or above SAE—number (percentage)	21	11 (52.4)	5 (23.8)	5 (23.8)
Patients with at least one SAE—number (percentage)	16	9 (56.2)	3 (18.8)	4 (25.0)
Patients with ≥ 2 SAE—number (percentage)	4	2 (50.0)	1 (25.0)	1 (25.0)
Type of SAE—number (percentage)				
Muco-cutaneous	2 (9.5)	1 (9.09)	0 (0.0)	1 (25.0)
COVID-19	4 (19.0)	3 (27.2)	0 (0.0)	1 (25.0)
Pneumonia	3 (14.3)	3 (27.2)	0 (0.0)	0 (0.0)
Hemoptysis	1 (4.8)	1 (9.09)	0 (0.0)	0 (0.0)
Urinary tract infection	9 (42.8)	1 (9.09)	5 (100.0)	3 (60.0)
Acute coronary syndrome	1 (4.8)	1 (9.09)	0 (0.0)	0 (0.0)
Pharyngitis	1 (4.8)	1 (9.09)	0 (0.0)	0 (0.0)
Mean number of injections prior to SAE^a^	4.45 ± 2.24	5.30 ± 2.20	3.20 ± 2.59	3.75 ± 0.96
Mean IgG count prior to SAE	9.37 ± 2.75	9.25 ± 3.49	8.73 ± 1.72	10.13 ± 0.64
HypoIgG—number (percentage)	6 (33.3)	5 (50.0)	1 (33.3)	0 (0.0)
Mean IgM count prior to SAE^a^	0.72 ± 0.46	0.60 ± 0.41	0.66 ± 0.38	1.05 ± 0.55
HypoIgM—number (percentage)	5 (29.4)	5 (50.0)	0 (0.0)	0 (0.0)
Mean CD19^+^ count prior to SAE^b^	9.62 ± 20.35	5.6 ± 12.33	1.00 ± 0.00	34.00 ± 46.67
Depleted prior to SAE (%)	11 (84.6)	9 (90.0)	1 (33.3)	1 (50.0)
Mean EDSS prior to SAE	4.32 ± 2.76	3.20 ± 2.30	6.80 ± 0.40	8.50 ± 0.00
Mean number of immunosuppressants prior to BCDT	1.13 ± 1.09	0.89 ± 0.78	2.67 ± 1.15	0.50 ± 0.58
Sex Female (%)	50	66.67	0	50
Disease duration—years	13.45 ± 9.60	11.71 ± 10.08	24.39 ± 5.12	9.19 ± 4.40
Age—years	44.59 ± 8.42	40.61 ± 7.40	47.62 ± 7.64	51.28 ± 7.28
Discontinued BCDT (percentage)	1 (6.25)	0	1 (33.33)	0

^a^Immunoglobulin IgG and IgM levels available for 17 events, of which 10 in relapsing category, 3 in secondary progressive and 4 in primary progressive category

^b^CD19^+^ counts available for 13 events, of which 10 in relapsing category, 3 in secondary progressive category and 2 in primary progressive category

Concerning biological data prior to SAE, the average mean absolute CD19^+^ count was 9.62 ± 20.35 cells/mm^3^, absolute IgM and IgG levels were 0.72 ± 0.46 and 9.37 ± 2.75, respectively. The overall percentage of sustained B-cell-depletion prior to SAE was 84.6% while hypoIgM was observed in 29.4% and 35.3% for hypoIgG. Patients with SAE had a significantly longer treatment duration (*p* = 0.02) when comparing to those without SAE. There were no differences in age at disease onset, sex ratio EDSS at baseline, number of immunosuppressants prior to BCDT, lymphocyte subset counts, lymphocytopenia, Ig isotype levels or hypogammaglobulinemia between the two groups. The results are summarized in supplemental Table 1. We did not perform predictive analysis due to the low number of patients in the group with SAE.

## Discussion

The results in this present study highlight that a majority of patients on BCDT show disease control at 18 months independent of their MS phenotype when considering NEDA-3 and MEDA status with brain and spinal cord analysis. We further suggest that sustained B-cell depletion observed in most of patients at 18 months is insufficient in predicting MS disease control. We show that long-term anti-CD20 therapies comes at the cost of significant treatment-related hypoIgG after 30 months of BCDT and infectious SAEs that are associated with longer treatment duration. Furthermore, prior treatment to BCDT should be taken into consideration given that prior LIT is associated with lower absolute IgG levels and hypoIgG in patients on long-term BCDT.

 Our study supports previous results regarding the efficacy of BCDT with a reduction in ARR at 12 and 18 months [22, 31–33]. We observed a considerable proportion of patients who were both NEDA-3 and MEDA at 12 and 18 months. No studies have analyzed NEDA-3 outcomes in BCDT including brain and spinal cord MRI studies, and MEDA outcomes have yet to be reported for patients on BCDT. Post-hoc analysis of OPERA I and II showed 72.2% of patients were NEDA-3 after brain MRI re-baseline between 24–96 weeks post-BCDT [34]. Our results taking into account brain and spinal cord MRI are similar considering that 82.8% of RMS patients in our study attained NEDA-3 at 18 months. The fact that we compared MRI imaging at this time-point to the re-baselined MRI provides a strength to our study, since an early re-baselined MRI may reflect persistent inflammatory activity prior to BCDT efficacy and thereby negatively impact NEDA-3 status achievement [34]. Indeed, we observed that failure of NEDA-3 and MEDA at 12 months was primarily due to MRI activity. At 12 months, it is possible that MRI activity may reflect the presence of new lesions prior to treatment initiation, or that new lesions may appear prior to treatment efficacy considering that anti-CD20 therapies have yet to reach its full efficacy during the first six months [34, 35]. MEDA has been shown to be associated with minimal risk for increased long-term disability in RMS patients on interferon beta or glatiramer acetate [30]; nevertheless, future studies will need to address if achieving MEDA status is sufficient to prevent long-term disability in patients on BCDT.

Age has been associated with greater inflammatory disease control [36]. A study by Cellerino et al. observed improved disease control in younger RMS patients with regards to NEDA-3 status at 24 months post-BCDT [36]. However, our study did not find an association with age, which may be due to the inclusion of PMS patients in our cohort who tend to show less clinical and radiological inflammatory activity at an older age. Additionally, lower NEDA at 12 months could be due to the comparison of the re-baselined MRI to the MRI prior to BCDT.

Our results support a growing body of literature that following absolute CD19^+^ B-cell counts post-BCDT is not sufficient, per se, to predict disease control [10, 14, 15, 27, 37]. While our study did not perform B-cell subset analyses, it has been shown that B-cell subsets post-BCDT tend to be more naive and transitional, and less towards a memory-B phenotype, which is thought to be implicated in MS pathogenesis [27, 37, 38]. Repopulation of CD27^+^ memory B-cells after rituximab remains low even at Week 52 post-infusion [38]. The slower repopulation kinetics of memory-B cells may also explain as to why early CD19^+^ B-cell repopulation in patient cohorts with either extended BCDT dosing intervals above the standard 6-month reinfusion or interruption do not show significant clinical worsening or new MRI lesions [10, 14, 15, 39, 40]. BCDT reinfusion based on CD27^+^ memory B-cells has been adopted by certain groups treating patients with neuromyelitis optica spectrum disorder, myasthenia gravis, as well as patients with MS [11, 16–19]. Nevertheless, randomized control trials and defined memory B-cell reconstitution cutoffs need to be properly established in MS in order to guide dosing intervals.

Given that NEDA-3 and MEDA criteria were not achieved in patients that showed sustained B-cell depletion, we also looked at other biological markers that could be predictive of MS disease control. Although it has been shown that CD20^+^ T lymphocytes enriched in the CD8^+^ T-cell compartment are proinflammatory and are present in periphery of MS patients [38, 41, 42], no T-cell lymphocyte subset was associated with MS disease control. Furthermore, it has been previously observed that disease control in patients treated by rituximab for rheumatoid arthritis (RA) was better in patients with IgM hypogammaglobulinemia [9]; however, we did not observe any Ig isotype as a predictor for disease control.

Over 50% of patients were hypoIgM at 30 months, while just 15% of patients were hypoIgG at this time point, similarly to a previous study investigating long-term rituximab treatment in MS and its effects on gammaglobulin levels [43]. Observational studies suggest that treatment-induced hypogammaglobulinemia in MS is associated with an increased risk of infection, although increased risk of SAE is debated [23, 43, 44]. In our study, SAEs while on long-term BCDT were infrequent, and our results are consistent with previous studies in autoimmune neurological and rheumatological diseases [20, 44–46]. Similar to previous studies, patients with SAE had a longer treatment duration than those without SAE, thus suggesting that cumulative long-term exposure to IV BCDT confers a risk of SAE in MS patients [23, 44]. Predictors of SAE, such as hypogammaglobulinemia, were not possible considering the low number of events over the study period.

Few studies have investigated the cumulative effect of previous DMTs prior to BDCT induction and their influence on biological parameters. DMTs such as fingolimod and dimethyl fumarate are known to induce lymphocytopenia, and have been shown to impact T-lymphocyte subsets and increase the risk of T-lymphocytopenia at baseline and up to 12 months after BCDT induction [25]. Our study confirms and extends the results of this former study, as we observed a significant decrease in the absolute CD45^+^, CD3^+^ and CD4^+^ counts and CD4^+^ lymphocytopenia until 24 months in patients having received prior LIT including fingolimod and dimethyl fumarate, but also cytotoxic immunosuppressive agents. Although we grouped all prior LIT together for analysis in this study, it should be highlighted that S1P receptor modulators (siponimod and fingolimod) included in the analysis differ mechanistically from the other LITs. Given that these treatments prevent the egress of lymphocytes from peripheral lymphoid tissue, this may therefore impact the findings in patients switching directly from an S1P receptor modulator.

In addition to differences in T-lymphocyte subsets, we also observed a significant decrease in absolute IgG levels at baseline and after 24 months in BCDT patients having received prior LIT. A higher proportion of patients with hypoIgG was also observed at 30 months in this population. It has been shown that cyclophosphamide treatment prior to rituximab in anti-neutrophil cytoplasm antibody associated vasculitis was associated with decreased serum IgG concentrations [47], yet concomitant use of methotrexate and rituximab in rheumatoid arthritis patients was observed to be a protective factor in the risk of developing hypogammaglobulinemia [48]. With regard to MS, prior fingolimod treatment has been shown to influence hypogammaglobulinemia [49], while Ig levels remain relatively stable in patients over at least 96 weeks of dimethyl fumarate [50]. MS patients treated with natalizumab have reduced Ig levels over time [49, 51], which may be attributed to impairment of B-cell maturation in the periphery [52]. In our study, it could be possible that prior LIT in BCDT treated patients exerts a synergistic effect on decreasing IgG levels at later BCDT cycles by accelerating the depletion of IgG producing mature B-cells in the peripheral blood. Nonetheless, the ultimate consequences of prior LIT exposure in relation to BCDT is not clear, considering we did not observe an increase in serious opportunistic infections or malignancies in our cohort. Further studies are needed in order to uncouple the role of prior LIT with respect to BCDT.

This study has several limitations, of which the retrospective and observational design, thereby limiting the possibility for collecting all adverse events throughout the study period. However, we focused on grade-3 or above SAEs, which are often documented, and ultimately reduced the likelihood of missing safety data. Furthermore, the monocentric design of our study most likely led to a limited sample size. The retrospective design of the study, inclusion of certain patients in randomized control trials, and loss to follow up, all contributed to missing biological data. Nevertheless, our clinic is an MS expert center with exhaustive clinical, biological and brain and spinal MRI activity in routine, and only 15% of patients lacked initial clinical or biological data. Of note, NEDA takes into account confirmed clinical progression, and therefore NEDA attainment could not be calculated for some patients given the lack of EDSS score at 12 and 18 months. Although we observed limited variations in perfusion intervals, most notably during the COVID-19 pandemic, we observed a significantly longer perfusion interval in comparing before and after March 2020. These results are unsurprising, given that we share space with elective orthopedic surgery, which was less active during the COVID-19 pandemic, and therefore our perfusion clinic was able to maintain clinical activity during this time since. Nevertheless, this difference in delay was clinically irrelevant with regards to the results.

Given the high proportion of patients with MS disease control after the first year of treatment, especially when considering less strict disease activity such as MEDA, it is tempting to suggest a possible opportunity to readapt BCDT at the 18-month time period. Furthermore, given the risk of developing significant treatment-induced hypoIgG at 30 months and the non-negligible risk of SAE occurring after a mean of 4 cycles of BCDT (i.e. 18-months), this time point may be useful in future studies that look to tailor anti-CD20 therapies. Rheumatological diseases have readapted their treatment strategy by a “treat-to-target approach” based on clinical activity [9], yet with respect to certain autoimmune neurological diseases the treatment strategy is based on biological parameters rather than new clinical activity [17, 18]. Our study suggests that sustained CD19^+^ B-cell depletion is insufficient to predict clinical or radiological disease control in MS patients, and therefore may not be a useful guide in order to aid neurologists in tailoring BCDT reinfusion in this patient population. These data highlight other variables that may need to be considered in the balance for BCDT tailoring, such as prior LIT. A randomized controlled trial would be useful in order to investigate a potential induction strategy followed by maintenance therapy, similar to other autoimmune diseases treated with BCDT.

### Supplementary Information

Below is the link to the electronic supplementary material.

**Supplemental figure 1.** Percentage lymphocytopenia and hypogammaglobulinemia in the patient cohort. Percentage of patients with lymphocytopenia and percentage of patients presenting with CD19+ depletion (A), as well as hypogammaglobulinemia (B), from baseline to 66 months post-BCDT. (B) Shows the percentage of patients with IgA, IgM and IgG hypogammaglobulinemia. Number of patients analyzed is detailed below each month. (TIFF 2296 KB)

Supplementary file2 (DOCX 18 KB)
