# Supplementary figures and images for: Assessing Sustained B-Cell Depletion and Disease Activity in a French Multiple Sclerosis Cohort Treated by Long-Term IV Anti-CD20 Antibody Therapy
Source: Neurotherapeutics. 2023 Oct 26;20(6):1707–22. doi: 10.1007/s13311-023-01446-5 (PMC10684468; doi:10.1007/s13311-023-01446-5)

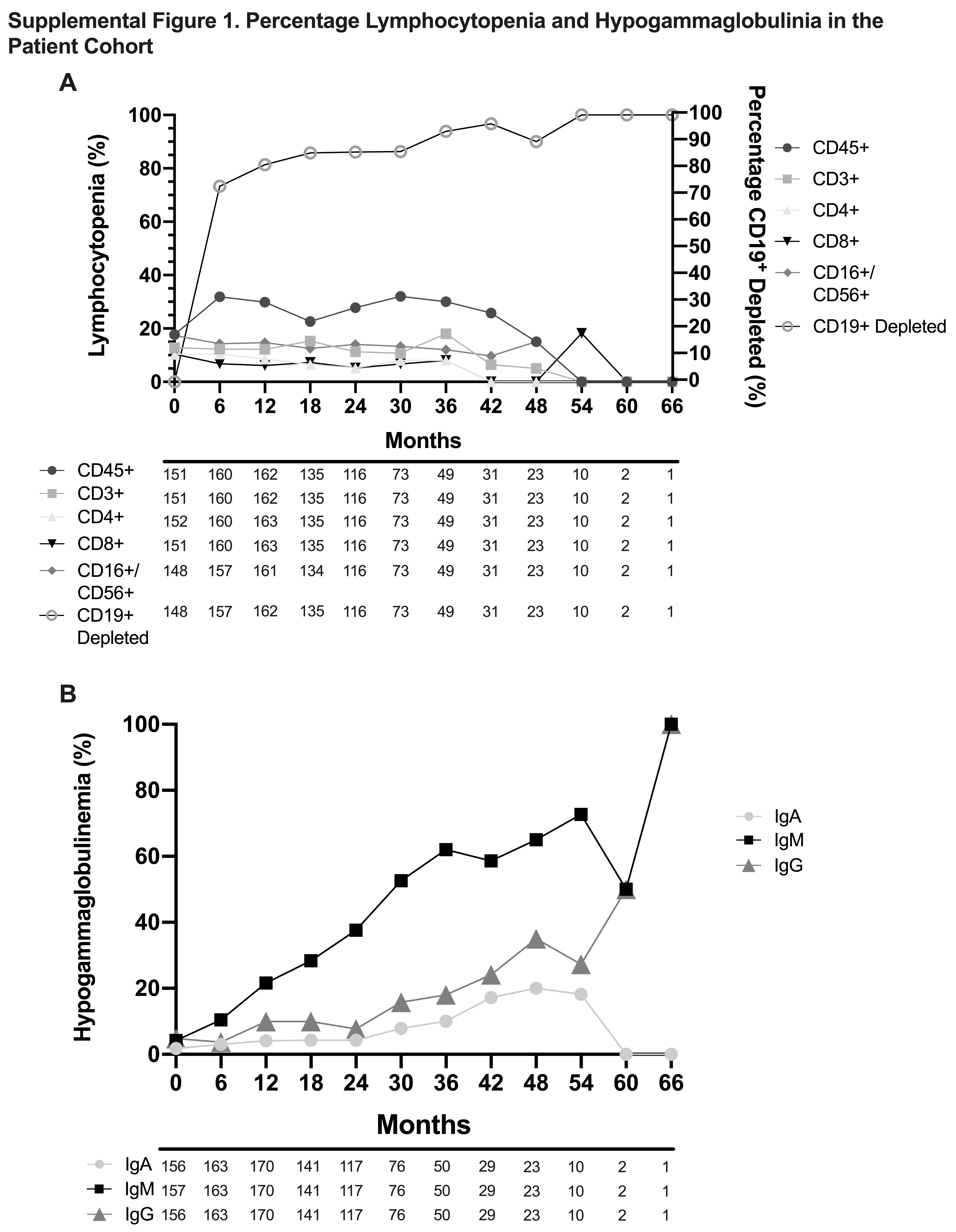

Supplement: Supplementary file 1 — Supplemental figure 1. Percentage lymphocytopenia and hypogammaglobulinemia in the patient cohort. Percentage of patients with lymphocytopenia and percentage of patients presenting with CD19+ depletion (A), as well as hypogammaglobulinemia (B), from baseline to 66 months post-BCDT. (B) Shows the percentage of patients with IgA, IgM and IgG hypogammaglobulinemia. Number of patients analyzed is detailed below each month. (TIFF 2296 KB) [file 13311_2023_1446_MOESM1_ESM.tiff]
