# Supplementary material for: Assessing Sustained B-Cell Depletion and Disease Activity in a French Multiple Sclerosis Cohort Treated by Long-Term IV Anti-CD20 Antibody Therapy
Source: Neurotherapeutics. 2023 Oct 26;20(6):1707–22. doi: 10.1007/s13311-023-01446-5 (PMC10684468; doi:10.1007/s13311-023-01446-5)
Supplement: Supplementary file 2 — Supplementary file2 (DOCX 18 KB) [file 13311_2023_1446_MOESM2_ESM.docx]

| **Supplemental Table 1. Baseline characteristics of patients with and without a serious adverse event prior to anti-CD20 therapy** | | | |
| --- | --- | --- | --- |
| **Characteristic** | **Patients with SAE (n=16)** | **Patients without SAE**  **(n=176)** | ***P-value*** |
| **Age at disease onset—years** | 42.4±8.48 | 42.6±11.9 | 0.936 |
| **Sex Ratio Female—number (%)** | 8 (50) | 112 (63.6) | 0.293 |
| **Disease duration—years** | 15.0±9.47 | 13.3±8.70 | 0.562 |
| **Median EDSS at baseline (range)*** | 4.25 (0-8) | 3.5 (0-7.5) | 0.107 |
| **Treatment duration—years** | 3.45±1.31 | 2.68±1.28 | **0.020** |
| **Median number of immunosuppressants prior to BCDT (range)** | 1.0 (0-6) | 1.0 (0-7) | 0.876 |
| **Mean total initial CD45^+^ Lymphocyte count—mm^3†^** | 1541±716.7 | 1735±729.8 | >0.999 |
| **CD3^+†^** | 1122±547.5 | 1324±750.5 | 0.634 |
| **CD4^+‡^** | 641±403.1 | 842.4±395.2 | 0.123 |
| **CD8^+†^** | 421.5±198.9 | 488±245.4 | 0.564 |
| **CD19^+¶^** | 210.1±126.8 | 246.1±192.2 | 0.649 |
| **CD16^+^CD56^+¶^** | 157.8±54.55 | 182.5±93.91 | 0.565 |
| **Number of patients with lymphocytopenia (%)** |  |  |  |
| **CD45^+†^** | 2 (18.2) | 27 (17.6) | >0.999 |
| **CD3^+†^** | 2 (18.2) | 19 (12.4) | 0.634 |
| **CD4^+‡^** | 3 (25.0) | 14 (9.1) | 0.111 |
| **CD8^+†^** | 2 (18.2) | 15 (9.8) | 0.318 |
| **CD19^+¶^** | 1 (10.0) | 18 (11.8) | >0.999 |
| **CD16^+^CD56^+¶^** | 1 (10.0) | 27 (17.8) | >0.999 |
| **Mean Ig levels—g/L** |  |  |  |
| **IgA^#^** | 2.11±0.79 | 2.08±0.77 | 0.949 |
| **IgG^^^** | 10.28±2.97 | 10.38±2.54 | 0.583 |
| **IgM^#^** | 1.14±0.47 | 1.11±0.57 | 0.519 |
| **Number of patients with hypogammaglobulinemia—number (%)** |  |  |  |
| **IgA^#^** | 0 (0.0) | 3 (1.96) | >0.999 |
| **IgG^^^** | 1 (7.69) | 7 (4.55) | 0.484 |
| **IgM^#^** | 0 (0.0) | 7 (4.58) | >0.999 |

| * A total number of 14 patients with EDSS scores in the patients with SAE and 162 EDSS scores in patients without SAE |
| --- |
| †Total number of patients with CD45+, CD3+ and CD8+ flow cytometry counts for patients with SAE is 11 and 153 for patients without SAE |
| ‡Total number of CD4+ flow cytometry counts for patients with SAE is 12 and 153 for patients without SAE |
| ¶Total number of CD19+ and CD16+CD56+ flow cytometry counts for patients with SAE is 10 and 151 for patients without SAE |
| #Total number of patients with IgM and IgA is 13 for patients with SAE is 13 and 154 for patients without SAE |
| ^Total number of patients with IgG is 13 for patients with SAE and 154 for patients without SAE |
